# Prevalence of sexual dysfunction and its association with fertility quality of life among infertile men in Henan, China

**DOI:** 10.1530/RAF-25-0195

**Published:** 2026-06-30

**Authors:** Zhongxin Wang, Xuan Su, Ruonan Li, Yifei Wang, Nurul Akmal Jamaludin, Hana Chen

**Affiliations:** ^1^ International Medical School, Management and Science University, Shah Alam, Malaysia; ^2^ School of Graduate Studies, Management and Science University, Shah Alam, Malaysia; ^3^ School of Nursing and Health, Henan University, Kaifeng, Henan, China; ^4^ Department of Community Medicine, Faculty of Medicine, University of Cyberjaya, Cyberjaya, Malaysia

**Keywords:** infertile men, sexual dysfunction (SD), fertility quality of life (FertiQoL), Arizona sexual experience scale (ASEX)

## Abstract

**Abstract:**

Infertility is a global public health issue, with male factors contributing to about 50% of cases. Yet, studies on fertility quality of life (FertiQoL), sexual dysfunction (SD), and their interconnection remain understudied in China, especially in Henan Province. This study aimed to assess the prevalence of SD, measure FertiQoL, and examine their association among infertile men in Henan. A cross-sectional study was conducted among infertile men aged 18–54 years, recruited via quota and purposive sampling. Validated instruments such as the FertiQoL and the Arizona Sexual Experience Scale (ASEX) were used. Data were analyzed using chi-square tests and multivariable regression analysis. A total of 319 participants completed the survey. Overall, 69.9% had poor FertiQoL, and 24.8% experienced severe SD. The mean overall FertiQoL score was 33.5 ± 24.4, with similar mean scores across the emotional (33.8 ± 24.9), social (34.3 ± 26.0), and treatment-related (32.5–33.8) domains. Severe SD was independently associated with poor FertiQoL (AOR = 4.34, 95% CI = 2.37–7.97). Longer infertility duration (>10 years), middle age (35–44 years), and ART failure were also independently associated with poor FertiQoL. SD and poor FertiQoL are prevalent among infertile men in Henan, with all FertiQoL domains markedly affected. This study fills regional data gaps and highlights the need for targeted psychosocial screening and interventions in infertility care. Addressing sexual health and emotional support may improve the overall quality of life and treatment outcomes for infertile men in Henan.

**Lay summary:**

This study shows that sexual problems significantly affect the quality of life in infertile men, highlighting the need for comprehensive support beyond medical treatment.

## Introduction

Infertility, defined by the World Health Organization (WHO 2023) as the failure to achieve clinical pregnancy after 12 months of regular unprotected intercourse, affects 8–12% of reproductive-age couples globally, representing a major public health challenge with profound physical, emotional, and social impacts (WHO 2023). Male factors independently account for 20–30% of infertility cases and contribute to 50% of combined male–female infertility (Agarwal *et al.* 2021), with the global prevalence of male infertility increasing by 76.9% between 1990 and 2019 (Huang *et al.* 2023). In China, the burden is equally severe: male infertility cases rose from 11.92 million in 1990 to 14.57 million in 2019 (Yu *et al.* 2023), and this upward trend is compounded by sociocultural pressures – such as traditional values emphasizing ‘family lineage’ and male reproductive responsibility – that exacerbate psychosocial distress among infertile men (Inhorn & Patrizio 2015).

Despite the high burden, research on the psychosocial dimensions of male infertility remains disproportionately focused on women (Song *et al.* 2021), leaving critical gaps in understanding male-specific experiences. Fertility quality of life (FertiQoL), a validated tool measuring infertility’s impact on emotional, relational, and social well-being (Boivin *et al.* 2011), and sexual dysfunction (SD) – a common comorbidity including erectile dysfunction, reduced libido, and orgasm difficulty – are two key outcomes rarely studied in tandem among Chinese male infertility patients, especially in underrepresented regions such as Henan Province.

Henan, China’s most populous province (≈99 million residents (Henan Provincial Bureau of Statistics 2024)), exhibits unique sociocultural contexts (e.g. stronger patriarchal norms and stigma around male infertility) that may amplify the association between SD and poor FertiQoL, yet no regional data exist. Existing Chinese studies are geographically limited: Abulizi *et al.* (2023) documented poor FertiQoL among infertile men in Xinjiang (Abulizi *et al.* 2023), and Gao *et al.* (2013) linked SD to psychological burden in a large Chinese male cohort (Gao *et al.* 2013), but neither addressed Henan’s specific needs. Globally, Coward *et al.* (2019) found erectile dysfunction correlated with lower FertiQoL in men undergoing fertility treatment, but cross-cultural generalizability to Chinese population, where infertility stigma is more pronounced (Dyer & Patel 2012), remains untested.

This study focuses on three sets of independent variables (IDVs) and their association with the dependent variable (DV: FertiQoL). First, SD (IDV1) is strongly linked to poor FertiQoL: Lotti & Maggi (2018) noted SD in infertile men triggers anxiety and self-esteem issues (Lotti & Maggi 2018), which directly reduce emotional and social well-being (key FertiQoL domains). Gao *et al.* (2013) further confirmed that Chinese infertile men with SD had 2.3-fold higher odds of psychological distress, a mediator of FertiQoL impairment. Second, sociodemographic factors (IDV2: age, education, and income) influence FertiQoL: Jimbo *et al.* (2022) showed men over 45 years have lower FertiQoL due to declining reproductive potential, while Damayanti *et al.* (2022) reported low income and limited education correlate with poor FertiQoL via reduced access to support services. Third, fertility-related factors (IDV3: infertility duration and ART failure) exacerbate distress: Lok *et al.* (2002) found ART failure doubles emotional distress in Chinese patients, and Huang *et al.* (2023) linked infertility duration >10 years to chronic stress, a known predictor of poor FertiQoL. ART failure is defined as failure to achieve clinical pregnancy after a full IVF/ICSI cycle, which is a common challenge in male infertility treatment, but its impact on quality of life remains unclear. By addressing Henan’s data gap and quantifying these IDV–DV relationships, this study fills a critical void in regional and global infertility research.

## Methods

### Study design and setting

A quantitative, observational, and cross-sectional study was conducted from December 2024 to January 2025 in Henan Province, China. Henan was selected due to its large population and limited FertiQoL data (Henan Provincial Bureau of Statistics 2024). Data were collected from fertility clinics across five regions (Northern, Central, Western, Southern, and Eastern Henan) to ensure geographic representation.

### Ethical considerations

Ethical approval was obtained from the Ethics Committee of Management and Science University and the participating fertility clinics in Henan where the study was conducted. Informed consent was obtained from all participants, and all data were anonymized to ensure confidentiality.

### Study participants and sampling

Chinese male patients aged 18–54 years, clinically diagnosed with infertility, and residing in Henan were included in the study. Participants who had chronic illnesses or comorbidities, are clinically diagnosed with mental disorders, are using medications that affect sexual function, had prior SD unrelated to infertility, and had recent major life stressors (e.g. divorce) were excluded from the study, as these factors may be confounders for the study’s outcome. Non-probability quota sampling (stratified by Henan’s five regions) and purposive sampling (recruitment from fertility clinics) were used to ensure representativeness (Gameiro *et al.* 2015). Quota proportions were based on Henan’s population census (Henan Provincial Bureau of Statistics 2024). Recruitment was conducted by trained research assistants during the initial consultation visit for male infertility at the three centers. A combination of quota sampling (stratified by age: 20–30 years, 31–40 years, ≥41 years) and purposive sampling (inclusion of patients with a confirmed infertility diagnosis of ≥1 year) was used to invite eligible participants. A total of 428 patients were approached, and 319 agreed to participate (response rate: 74.5%). To assess the selection bias, we compared baseline characteristics (age, infertility duration, education level, and monthly income) between participants (*n* = 319) and non-participants (*n* = 109). No statistically significant differences were observed between the two groups (all *P* > 0.05), suggesting a low risk of selection bias related to these key variables.

### Study instruments and data collection

Data were collected via an anonymous, validated self-administered questionnaire in Chinese language. It comprised 48 items and three sections: Section A – sociodemographic profile and fertility-related issues (7 items), Section B – Arizona Sexual Experience Scale (ASEX) (5 items), and Section C – FertiQoL (36 items) (McGahuey *et al.* 2000, Li *et al.* 2019). The questionnaires were distributed in clinics. Each survey allowed only one response per person. The time taken to complete the survey was about 15–20 min.

The sociodemographic and fertility-related issues section captured age; education level: categorical variable (reference group: bachelor’s degree or above; comparison groups: primary school, junior high school, and senior high school/college); monthly income: categorical variable (low, middle, and high); infertility duration: continuous variable (measured in years); and ART failure (yes/no).

The ASEX is a five-item tool measuring sexual desire, arousal, penile erection, orgasm, and satisfaction (McGahuey *et al.* 2000). Each item is scored 1–6 (1 = normal, 6 = severe impairment). Total scores ≥19 indicate severe SD. The Chinese version has Cronbach’s *α* = 0.91.

The FertiQoL tool consists of 36 items with two modules, namely, the Core FertiQoL (24 items, with emotional, mind–body, relational, and social subscales) and the treatment FertiQoL (10 items, treatment environment, and treatment tolerability) (Boivin *et al.* 2011). Each item is scored 0–4, with raw scores converted to scaled scores (0–100) using (raw score × 25)/number of items. Lower scores indicate poorer quality of life. A meaningful classification of FertiQoL scores was derived using a quartile split. The observed score range (maximum–minimum) was divided into four equal segments, and the third quartile (Q3) was used as the cutoff point to indicate better FertiQoL. Using the data in this study, the cutoff point of <62 indicates poor FertiQoL and ≥62 indicates good FertiQoL. The Chinese version has Cronbach’s *α* = 0.89 (Li *et al.* 2019).

### Statistical analysis

Data were cleaned and analyzed using the SPSS version 26.0. Descriptive statistics (frequency, percentage, mean, and standard deviation) were used to summarize sociodemographic data, SD prevalence, and FertiQoL scores. Bivariate analysis using the chi-square test was performed to examine the associations between sociodemographic characteristics, fertility-related issues, and SD with FertiQoL. Multivariable logistic regression analysis was conducted to identify predictors of poor FertiQoL (outcome variable), adjusted for age, education level, monthly income, infertility duration, ART failure, and SD. A *P* < 0.05 was considered statistically significant.

For multivariable logistic regression models, covariates were initially selected based on a *P* < 0.25 in univariate analyses (age, infertility duration, education level, monthly income, and ART failure status). A backward stepwise elimination procedure (*α* = 0.05 for retention) was used to finalize the model. Robustness checks included i) multicollinearity assessment using the variance inflation factor (VIF); all VIF values were <2, indicating no multicollinearity; ii) exploration of interaction terms (infertility duration × education level; monthly income × ART failure status), none of which were statistically significant (all *P* > 0.05); and iii) sensitivity analysis comparing the stepwise model with a full model (including all initial covariates); core associations remained consistent, confirming model robustness.

## Results

### Sociodemographic and fertility-related characteristics

A total of 319 participants completed the survey. Table 1 presents the sociodemographic and fertility-related characteristics of the participants. 36.1% were 35–44 years old, 41.7% had tertiary education, 60.5% had low monthly income, 53.9% were married, and 67.1% were employed. The majority of the participants had infertility duration of 6 years or more (80.5%), and 68.0% had experienced ART failure.

**Table 1:** Sociodemographic and fertility-related characteristics of participants (*n* = 319).

Variables	Values, *n* (%)
Age group	
18–34 years	101 (31.7)
35–44 years	115 (36.1)
45–54 years	103 (32.3)
Education level	
Primary school or below	76 (23.8)
Secondary or vocational	110 (34.5)
Tertiary	133 (41.7)
Monthly income (RMB)	
Low	193 (60.5)
Middle	59 (18.5)
High	67 (21.0)
Marital status	
Single	45 (14.1)
Married	172 (53.9)
Divorced or separated	102 (32.0)
Employment status	
Employed	214 (67.1)
Unemployed	105 (32.9)
Infertility duration	
1–5 years	62 (19.4)
6–10 years	128 (40.1)
>10 years	129 (40.4)
ART failure history	
Yes	217 (68.0)
No	102 (32.0)

### Sexual dysfunction (SD)

**Figure 1:**
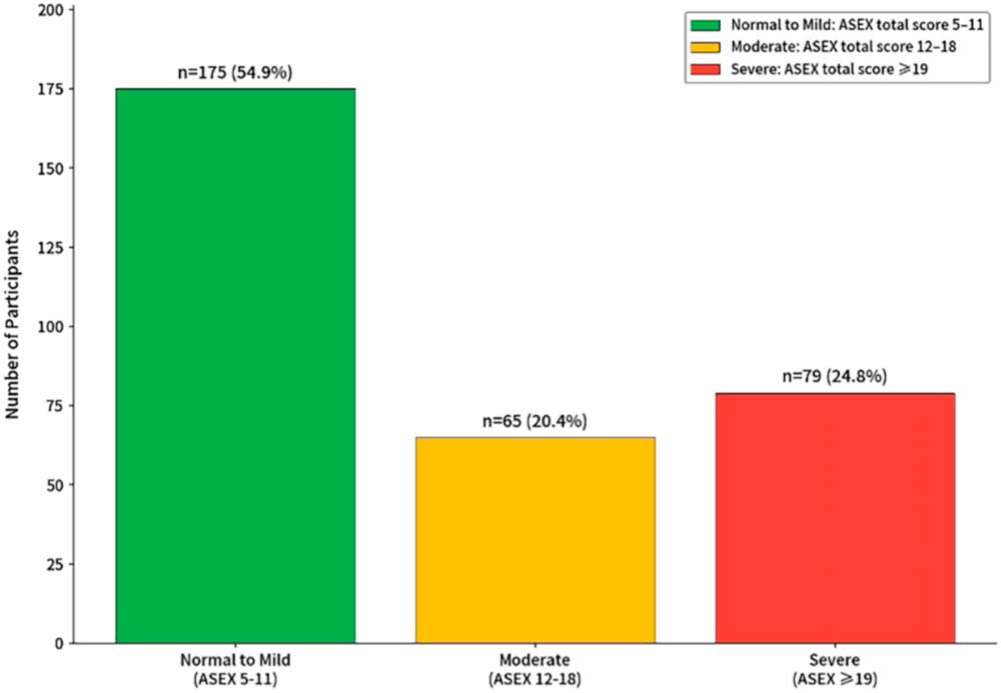
SD severity distribution of participants.

**Figure 2:**
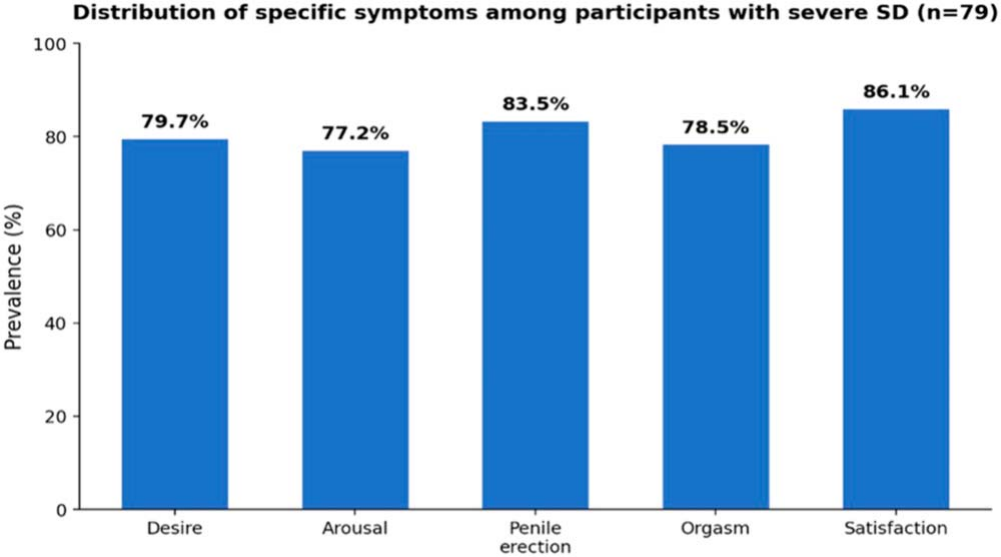
Distribution of specific symptoms in patients with severe SD (*n* = 79).

The participants’ ASEX scores ranged from 5 to 28 (mean = 13.6 ± 5.9). Severe SD (score ≥19) was observed in 24.8% (*n* = 79) of participants (Fig. 1). Within the severe SD subgroup, dysfunction was prevalent across all five ASEX domains, with the highest prevalence observed for satisfaction (86.1%) and penile erection difficulty (83.5%) (Fig. 2).

### Fertility quality of life (FertiQoL) and associated factors

**Figure 3:**
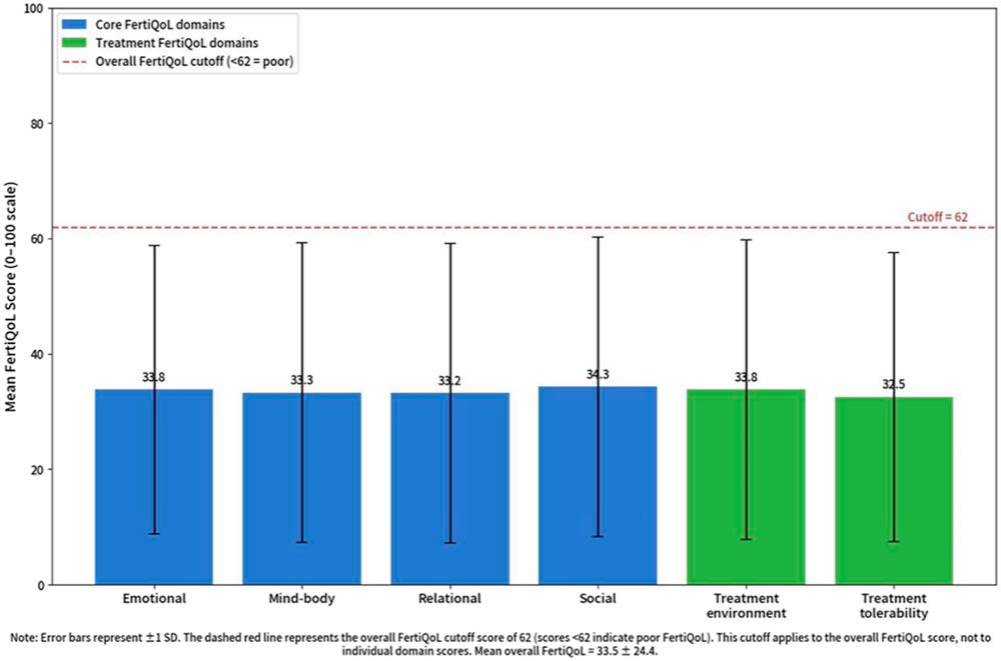
FertiQoL domain scores.

Figure 3 presents the domain scores of the FertiQoL. The mean overall FertiQoL score was 33.5 ± 24.4. The core module comprised four domains: emotional (33.8 ± 24.9), mind–body (33.3 ± 26.3), relational (33.2 ± 24.9), and social (34.3 ± 26.0). The treatment environment and treatment tolerability subscales recorded mean scores of 33.8 ± 25.8 and 32.5 ± 25.6, respectively. About 70% of the participants (*n* = 223, 69.9%) had poor FertiQoL (score < 62).

Table 2 presents the univariate logistic regression analysis, examining factors associated with poor FertiQoL (score < 62) among infertile men in Henan Province. Factors associated with poor FertiQoL based on a cutoff score of <62 were first analyzed using univariate logistic regression. Variables with a *P*-value of <0.25 in univariate analysis were selected as candidates for the multivariable logistic regression model (Table 3). The threshold of *P* < 0.25 is a widely used screening criterion in epidemiological research to avoid premature exclusion of potentially important confounders.

**Table 2:** Univariate logistic regression analysis of factors associated with poor FertiQoL (score < 62) among infertile men in Henan Province (*n* = 319).

Variable	OR	95% CI	*P-*value
Monthly income (low vs high)	1.357	1.018–1.809	0.037
Infertility duration (per year increase)	2.133	1.489–3.056	<0.001
Severe SD (ASEX score ≥19, yes vs no)	3.629	2.127–6.192	<0.001
Age (per year increase)	1.338	0.988–1.812	0.059
Education level (primary/secondary vs tertiary)	1.071	0.79–1.452	0.66
ART failure (yes vs no)	1.427	0.863–2.361	0.166

OR, crude odds ratio; 95% CI, 95% confidence interval.

Poor FertiQoL defined as overall FertiQoL score < 62. For continuous variables (age and infertility duration), OR represents the change in odds per unit increase. For categorical variables, reference categories are indicated in parentheses. Variables with *P <* 0.25 were selected as candidates for multivariable analysis (Table 3).

**Table 3:** Multivariable logistic regression analysis of factors associated with poor FertiQoL (score < 62) among infertile men in Henan Province (*n* = 319). All variables with *P* < 0.25 in univariate analysis (Table 2) were entered into the model. A backward stepwise elimination procedure (*α* = 0.05) was used for model selection. Multicollinearity was assessed using VIF (all values <2). Goodness of fit was indicated by the Hosmer–Lemeshow test (*P* = 0.668), and the model correctly classified 85.6% of cases. Only variables retained in the final model after stepwise elimination are shown with AOR values; variables not reaching statistical significance (*P* ≥ 0.05) in the final model are included to show the full set of candidate variables.

Variables	AOR	95% CI		*P*-value
		Lower	Upper	
Severe SD				
No	Ref			
Yes	4.342	2.366	7.967	<0.001
Age group				
18–34 years	Ref			
35–44 years	2.059	1.024	4.142	0.043
45–54 years	1.238	0.585	2.623	0.577
Education level				
Primary school or below	Ref			
Secondary	1.061	0.518	2.172	0.872
Tertiary	0.314	0.083	1.182	0.087
Monthly income				
Low	Ref			
Middle	2.264	0.609	8.413	0.223
High	6.677	1.778	25.072	0.005
Infertility duration				
1–5 years	Ref			
6–10 years	2.884	1.075	7.737	0.035
>10 years	4.553	1.706	12.148	0.002
ART failure				
No	Ref			
Yes	1.965	1.097	3.521	0.023

AOR, adjusted odds ratio; 95% CI, 95% confidence interval; Ref, reference categories.

As presented in Table 2, univariate logistic regression analysis showed that monthly income (OR = 1.357, 95% CI: 1.018–1.809, *P* = 0.037), longer infertility duration (OR = 2.133, 95% CI: 1.489–3.056, *P* < 0.001), and severe SD (OR = 3.629, 95% CI: 2.127–6.192, *P* < 0.001) were significantly associated with poor FertiQoL. Age showed a borderline association (OR = 1.338, 95% CI: 0.988–1.812, *P* = 0.059), while education level (OR = 1.071, 95% CI: 0.79–1.452, *P* = 0.66) and ART failure (OR = 1.427, 95% CI: 0.863–2.361, *P* = 0.166) did not reach statistical significance in the univariate analysis but were retained as candidate covariates for multivariable modeling.

Severe SD was independently associated with poor FertiQoL (AOR = 4.342, 95% CI: 2.366–7.967, *P* < 0.001) after adjusting for confounders in the multivariable model (Table 3).

After adjusting for all covariates in the multivariable model, several factors were independently associated with poor FertiQoL. Compared with the reference groups, severe SD (AOR = 4.342, 95% CI: 2.366–7.967, *P* < 0.001), middle age (35–44 years vs 18–34 years: AOR = 2.059, 95% CI: 1.024–4.142, *P* = 0.043), high monthly income (vs low income: AOR = 6.677, 95% CI: 1.778–25.072, *P* = 0.005), longer infertility duration (6–10 years vs 1–5 years: AOR = 2.884, 95% CI: 1.075–7.737, *P* = 0.035; >10 years vs 1–5 years: AOR = 4.553, 95% CI: 1.706–12.148, *P* = 0.002), and ART failure (AOR = 1.965, 95% CI: 1.097–3.521, *P* = 0.023) were independently associated with poor FertiQoL. Older age (45–54 years vs 18–34 years) was not statistically significant in the adjusted model (*P* = 0.577).

**Figure 4:**
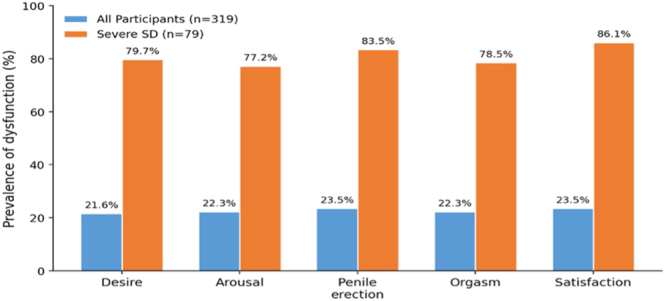
Comparison of the proportion of participants reporting severe impairment (ASEX item score ≥4) across sexual function domains between all participants (*n* = 319) and those with severe sexual dysfunction (ASEX total score ≥19; *n* = 79).

Figure 4 presents the prevalence of dysfunction across the five ASEX domains (desire, arousal, penile erection, orgasm, and satisfaction) for all participants (*n* = 319) and for the severe SD subgroup (*n* = 79). Among all participants, the prevalence of dysfunction across the five domains was relatively similar, ranging from 21.6% (desire) to 23.5% (penile erection and satisfaction). Within the severe SD subgroup, dysfunction was substantially more common across all five domains, with the highest prevalence observed for satisfaction (86.1%) and penile erection (83.5%), followed by desire (79.7%), orgasm (78.5%), and arousal (77.2%).

## Discussion

This cross-sectional study is the first to explore sexual dysfunction (SD), fertility quality of life (FertiQoL), and their correlates among 319 infertile men in Henan Province – China’s most populous region with distinct sociocultural pressures on male reproductive health. Below is a focused analysis of the core findings, grounded in targeted literature to avoid redundancy. Given the cross-sectional design, all associations reported below should be interpreted as correlational; the temporal sequence and causal direction of these relationships cannot be determined from the present data.

Severe SD (ASEX score ≥ 19) was observed in 24.8% of participants, a rate within the global range (12–31% in reproductive-age men; Lotti & Maggi 2018) and broadly in line with the 21.5% reported in a national Chinese infertile male cohort (Gao *et al.* 2013). Across the five ASEX domains, the prevalence of dysfunction in the overall sample was relatively even (21.6–23.5%), with penile erection difficulty and reduced satisfaction being the most commonly reported. Within the severe SD subgroup, dysfunction was substantially more prevalent across all five domains (77.2–86.1%), suggesting that severe SD reflects broad rather than domain-specific impairment in this population. Traditional sociocultural norms in Henan, which tie male sexual function to ‘masculine identity’ and ‘family lineage’ (Dyer & Patel 2012), may add psychological weight to these symptoms, as they can be perceived as ‘failure to fulfill marital and familial duties’. The ASEX score’s high reliability in Chinese populations (Cronbach’s *α* = 0.91) supports the validity of these prevalence estimates.

About 70% (69.9%) of participants had poor FertiQoL (score < 62), with consistently low mean scores across the emotional (33.8 ± 24.9) and social (34.3 ± 26.0) domains – aligning with findings from Xinjiang’s infertile men (Abulizi *et al.* 2023) but reflecting more pronounced distress in Henan. The emotional domain’s low score may be consistent with a hypothesized cycle in which SD, reduced self-esteem, and emotional distress co-occur and potentially reinforce one another (Lotti & Maggi 2018); however, the cross-sectional design of the present study cannot establish this directionality. Such patterns are also consistent with the sociocultural pressures described above.

The social domain’s score (34.3 ± 26.0) is markedly lower than global averages (typically >55 in non-clinical infertile cohorts) (Boivin *et al.* 2011), likely due to stigma-related social withdrawal. In Chinese cultural contexts, male infertility challenges traditional ‘provider’ and ‘ancestor-continuator’ roles (Inhorn & Patrizio 2015), a context in which some men may avoid social interactions to evade judgment, which could contribute to lower social FertiQoL. Severe SD was independently associated with poor FertiQoL (AOR = 4.342, 95% CI = 2.366–7.967) after adjusting for confounders (Coward *et al.* 2019).

In the multivariable model, severe SD, middle age (35–44 years), high monthly income, longer infertility duration (6–10 years and >10 years), and ART failure were independently associated with poor FertiQoL. Older age (>45 years) was not statistically significant in the adjusted model, contrasting with reports linking advanced reproductive age to declining reproductive potential and heightened ‘fertility failure’ stress (Jimbo *et al.* 2022), and may indicate that age-related psychological burden in this clinic-based Henan sample is overshadowed by other concurrent factors, such as treatment history and economic context. Notably, monthly income showed an unexpected pattern: compared with low-income participants, those with high income had higher odds of poor FertiQoL (AOR = 6.677, 95% CI: 1.778–25.072, *P* = 0.005), which differs from earlier reports linking low income to poor FertiQoL in other infertile populations (Damayanti *et al.* 2022). One possible interpretation is that higher-income men in Henan may face stronger career and family expectations, more pronounced social comparison, and greater perceived pressure to fulfill the ‘provider’ role, all of which can contribute to psychological burden when fertility is compromised. This finding is exploratory and requires confirmation in larger and more economically diverse samples. Long infertility duration (>10 years; AOR = 4.553) and ART failure (AOR = 1.965) may relate to prolonged stress exposure: Huang *et al.* (2023) reported an association between infertility >10 years and chronic stress that is associated with reduced emotional regulation, while ART failure (experienced by 68.0% of participants) is correlated with increased hopelessness (Lok *et al.* 2002). Limited rural ART access in the region may add to this burden and is consistent with the relatively low treatment tolerability scores observed (32.5 ± 25.6) (Henan Provincial Bureau of Statistics 2024).

Globally, Boivin *et al.* (2011) identified emotional and social domains as most vulnerable to infertility distress, a pattern observed here. Gao *et al.* (2013) linked SD to psychological burden in Chinese infertile men, and the present study extends this evidence to Henan.

In line with Coward *et al.* (2019), who reported erectile dysfunction as a key SD predictor of poor FertiQoL in Australian men, penile erection difficulty (83.5%) was among the most prevalent symptoms in the severe SD subgroup of the present study, alongside reduced satisfaction (86.1%). The relatively even distribution of dysfunction across the five ASEX domains suggests that, in this Henan cohort, severe SD tends to manifest as broad sexual impairment rather than a single dominant symptom, which may align with local cultural values that frame sexual function as integral to ‘marital harmony’ (Dyer & Patel 2012) – underscoring the need for region-specific, multi-domain interventions.

### Limitations

First, the use of quota and purposive sampling from tertiary fertility clinics in Henan Province limits the external validity of our findings. Our results are generalizable only to male infertility patients seeking care at tertiary hospitals in central China and cannot be extended to non-clinic-based populations or patients in other regions. Second, the cross-sectional design prevents us from establishing causal relationships between variables, and recall bias may have influenced self-reported outcomes (e.g. infertility duration and sexual function). Third, reliance on self-reported data (e.g. SD may be underreported due to stigma) (Elnazer & Baldwin 2020) and exclusion of biological markers (e.g. sperm quality) may confound results. Future studies should use longitudinal designs and probability sampling to validate findings.

## Implications

### Academic implications

This study fills a regional gap by providing data on male infertility’s psychosocial impacts in Henan, a populous but understudied region. It confirms the cross-cultural relevance of SD-FertiQoL associations (Coward *et al.* 2019) and highlights the need for culturally adapted interventions (e.g. addressing stigma around male infertility in Chinese contexts).

### Clinical implications

Healthcare providers should integrate SD and FertiQoL assessments into male infertility care. Targeted interventions (e.g. cognitive–behavioral therapy for SD and support groups for emotional distress) could improve outcomes (Cousineau & Domar 2007). For example, men with severe SD or long infertility duration may benefit from specialized counseling.

### Mechanistic implications

It is important to note that this study uses a cross-sectional design, which precludes causal inferences and temporal ordering of associations. The observed relationships between infertility, sexual dysfunction, and quality of life require confirmation in prospective cohort studies.

### Policy and social implications

Findings inform public health policies, such as expanding insurance coverage for ART and sexual health services in Henan. Raising awareness about male infertility’s psychosocial impacts can reduce stigma (Dyer & Patel 2012). In addition, workplace policies (e.g. flexible hours for treatment) may alleviate stress for employed participants.

### Global relevance

Results provide a framework for studying infertility in low- and middle-income regions with similar cultural dynamics (e.g. emphasis on family lineage). They also support the WHO’s (2023) call to prioritize male involvement in reproductive health.

## Conclusion

Based on the investigation of male infertility patients in Henan Province, this study found that male infertility is not only associated with a high prevalence of severe sexual dysfunction, but also significantly reduces the quality of life of patients. Socioeconomic status and failure of ART treatment are important influencing factors for the poor quality of life of male infertility patients. The results of this study enrich the evidence on the physical and mental effects of male infertility, suggesting that in clinical practice, attention should be paid to the sexual function and mental health of infertile men, especially those with low socioeconomic status and ART failure. Targeted psychological intervention and social support should be implemented to improve their overall quality of life.

## Declaration of interest

The authors declare that there is no conflict of interest that could be perceived as prejudicing the impartiality of the work reported.

## Funding

This research did not receive any specific grant from any funding agency in the public, commercial, or not-for-profit sector.

## Author contribution statement

WZ, HC, and NA conceptualized and designed the study. SX and LR performed validation and reliability of the questionnaire. WY and LR collected the data. WZ, SX, and HC conducted the statistical analysis and interpretation of the findings. WZ, LR, and WY wrote the initial draft. WZ and HC wrote the final manuscript. NA critically reviewed and finalized the manuscript. All authors read and approved the final version of this manuscript.

## Data availability

The data of this study are available from the corresponding author upon reasonable request.
